# Nonlocal Means-Based Denoising for Medical Images

**DOI:** 10.1155/2012/438617

**Published:** 2012-02-20

**Authors:** Ke Lu, Ning He, Liang Li

**Affiliations:** ^1^College of Computing & Communication Engineering, Graduate University of Chinese Academy of Sciences, Beijing 100049, China; ^2^School of Information, Beijing Union University, Beijing 100101, China

## Abstract

Medical images often consist of low-contrast objects corrupted by random noise arising in the image acquisition process. Thus, image denoising is one of the fundamental tasks required by medical imaging analysis. Nonlocal means (NL-means) method provides a powerful framework for denoising. In this work, we investigate an adaptive denoising scheme based on the patch NL-means algorithm for medical imaging denoising. In contrast with the traditional NL-means algorithm, the proposed adaptive NL-means denoising scheme has three unique features. First, we use a restricted local neighbourhood where the true intensity for each noisy pixel is estimated from a set of selected neighbouring pixels to perform the denoising process. Second, the weights used are calculated thanks to the similarity between the patch to denoise and the other patches candidates. Finally, we apply the steering kernel to preserve the details of the images. The proposed method has been compared with similar state-of-art methods over synthetic and real clinical medical images showing an improved performance in all cases analyzed.

## 1. Introduction

Medical images obtained from Magnetic Resonance Imaging (MRI) and Computed Tomography (CT) and Ultrasound imaging (US) are the most common tools for diagnosis. These images are often affected by random noise arising in the image acquisition process. The presence of noise not only produces undesirable visual quality but also lowers the visibility of low-contrast objects. Image denoising is one of the classical problems in digital image processing. As a primary basis image processing procedure, noise removal has been extensively studied and many denoising schemes have been proposed, from the earlier smoothing filters and frequency domain denoising methods to the lately developed wavelet- [1–5], curvelet- [6], and ridgelet- [7] based methods, sparse representation [8] and K-SVD [9] methods, shape adaptive transform [10], bilateral filtering [11], NL-means based methods [12, 13], and more recently proposed nonlinear variational methods like the total variation minimization [14–16]. With the rapid development of modern digital imaging devices and their increasingly wide applications in our daily life, there are increasing requirements of new denoising algorithms for higher image quality. Particularly, in medical imaging, denoising is challenging since all kinds of noise cannot be easily modeled and are known to be tissue dependent, such as ultrasound images. Although noise gives an image a generally undesirable appearance, the most significant factor is that noise can cover and reduce the visibility of certain features within the image. The presence of noise gives an image a mottled, grainy, textured, or snowy appearance. In the imaging process, the energy of the high-frequency waves is partially reflected and transmitted at the boundaries between tissues having different acoustic impedances. Nevertheless, the diagnosis quality is often low and reducing speckle while preserving anatomic information is necessary to delineate reliably and accurately the regions of interest. Recently, it has been demonstrated that image patches are relevant features for denoising images in adverse situations. The related methodology can be adapted to derive a robust filter for medical images. Accordingly, in this paper we introduce a novel restoration scheme for medical images, inspired from the NL-means approach introduced by Buades et al. [12] to denoise 2D natural images corrupted by an additive white Gaussian noise.

 The rest of this paper is organized as follows. The noise distribution and estimation in medical images are depicted in Section 2.1. The brief description of NL-means algorithm is discussed in Section 2.2 while the improved NL-means algorithm and the denoising performance under Rician noise are analyzed Section 2.3. The supporting experimental results of improved NL-means algorithm compared to other denoising methods under various conditions are illustrated in Section 3. Finally, concluding remarks are given in Section 4.

## 2. Improved NL-Means Denoising Method

### 2.1. Noise Distribution and Estimation in Medical Images

The most MR images acquired in the Fourier domain are characterized by a zero-mean Gaussian probability density function (PDF). After the inverse Fourier transform, the noise distribution in the real and imaginary components will still be Gaussian due to the linearity and the orthogonality of the Fourier transform. However, due to the subsequent transform to a magnitude image, the noise distribution will be no longer Gaussian but Rician distributed. For an MR magnitude image defined on a discrete grid *Ω*, *M* = {*m*_*i*_ | *i* ∈ *Ω*}, then the PDF of *m*_*i*_ is

(1) $$\begin{array}{c} p\left(m_{i}\;|\;A,\sigma \right)=\frac{m_{i}}{\sigma^{2}}e^{-(m_{i}^{2}+A^{2}/2\sigma^{2})I_{0}(Am_{i}/\sigma^{2})\varepsilon (m_{i})}, \end{array}$$

where *I*_0_(·) is the 0th-order modified Bessel function of the first kind and *ε*(·) is the Heaviside step function. *σ* ^2^ denotes the variance of the Gaussian noise in the complex MR data, which can be independently estimated. When the underlying intensity *A* equals zero, the Rician PDF simplifies to a Rayleigh distribution:

(2) $$p\left(m_{i}\;|\;A,\sigma \right)=\frac{m_{i}}{\sigma^{2}}e^{-(m_{i}^{2}/2\sigma^{2})\varepsilon (m_{i})}.$$

**Figure 1:**
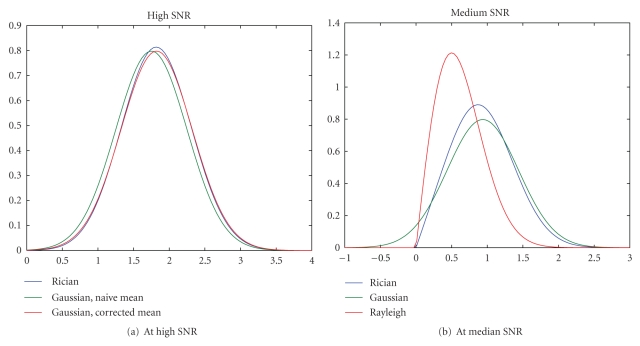
At high SNR, Rician data is approximately Gaussian. At low-medium SNR, neither Gaussian nor Rayleigh is a great approximation.

At high SNR, the Rician PDF approaches to a Gaussian PDF with a mean *A* and variance *σ* ^2^ (see Figure 1):

(3) $$p\left(m_{i}\;|\;A,\sigma \right)=\frac{1}{\sqrt{2\pi \sigma^{2}}}e^{-({\left(m_{i}-A\right)}^{2}/2\sigma^{2})\varepsilon (m_{i})}.$$

That is, Rician noise in magnitude MR images behaves like Gaussian distributed when SNR is high and Rayleigh distributed for low SNR.

 Now we discuss how to measure the noise variance from an MR image without the need for high SNR regions or a background region.

 Let *m*_1_, *m*_2_^  ^  ,…, *m*_*n*_ be the *n* Rician distributed magnitude data points, and region of constant signal intensity is *A*. Then the joint PDF of the observations is

(4) $$p\left(\left\{m_{i}\right\}\;|\;A,\sigma \right)=\overset{n}{\underset{i=1}{\Pi }}\frac{m_{i}}{\sigma^{2}}e^{-{(m}_{i}^{2}+A^{2})/2\sigma^{2}}I_{0}\left(\frac{Am_{i}}{\sigma^{2}}\right),$$

where {*m*_*i*_} are the magnitude variables corresponding to the magnitude observations *m*_*i*_. The maximum likelihood (ML) estimate of *A* and *σ* is then found from the global maximum of log⁡*L*:

(5) $${\hat{A}}_{\text{ML}}=\overset{n}{\underset{i=1}{\sum }}\ln\left(\frac{m_{i}}{\sigma_{2}}\right)-\overset{n}{\underset{i=1}{\sum }}\frac{m_{i}^{2}+A^{2}}{2\sigma^{2}}+\overset{n}{\underset{i=1}{\sum }}\ln I_{0}\left(\frac{Am_{i}}{\sigma^{2}}\right).$$

Since the noise is estimated from the available piecewise constant regions in the image, this estimation neither depends on the image background nor on the SNR of the image.

### 2.2. NL-Means Filter

We focus on the problem of denoising: an observed image *Y* is assumed to be a noisy version of an unobserved image *f* corrupted by white Gaussian noise. Let *Ω* ⊂ *Z* ^2^ be the indexing set of the pixels. For any pixel *x* ∈ *Ω*,

(6) $$\begin{array}{c} Y\left(x\right)=f\left(x\right)+\varepsilon \left(x\right) \end{array},$$

where *ε* is a centered Gaussian random variable with known variance *σ* ^2^ and the noise components *ε*(*x*) are independent. For each pixel the output of the procedure is a weighted average of the whole image. The weights used are selected using a “metric” which determines whether two pixels are similar or not. The core idea of the NL-means is to create a metric governed by patches surrounding each pixel, regardless of their position, that is, nonlocal in the image space. For a fixed (odd) width *p*, a patch *P*_*x*_ is a subimage of width *p*, centered around the pixel *x*, and the NL-means estimator of *f*(*x*) is:

(7) $$\hat{f}\left(x\right)=\frac{\sum_{x\prime \in \Omega }w\left(x,x\prime \right)Y\left(x\prime \right)}{\sum_{x\prime \in \Omega }w\left(x,x\prime \right)},$$

where *w*(*x*, *x*′) = exp⁡(−||*P*_*x*_−*P*_*x*′_||_2,*a*_^2^/2*h* ^2^), which measures the proximity between patches. *h* > 0 is the bandwidth, which has a smoothing effect and plays the same role as the bandwidth for kernel methods in statistics. The larger the bandwidth is, the smoother the image becomes. ||·||_2,*a*_ is a weighted Euclidean norm in *R* ^|*P*|^(|*P*| = *p* ^2^) using the Gaussian kernel, *a* controlling the concentration of the norm around the central pixel. The denominator is a normalizing factor ensuring the weights sum to one. The patch size *P* is generally chosen equal to 5, 7, or 9. From the patch estimator, it is possible to recover a pixel estimator by reprojection.

 In the following, the proposed filter is realized in three steps: (a) finding the image patches similar to a given patch; (b) applying the Rician estimation on the 3D block; (c) collaborative adaptive filtering.

### 2.3. Adaptation to Rician Noise Denoising Model

In case of Rician noise, there is no closed form for the ML estimate of the true signal *μ* given *n* such measures *x*_*i*_. However, the even order moments of the Rician law have very simple expressions. In particular, the second-order moment is *E*(*X*_*i*_^2^) = *μ* ^2^ + 2*σ* ^2^ where *σ* ^2^ is the variance of the Gaussian noise of MRI data. The measured value of *x*_*i*_^2^ (and that of *x*_*i*_) is thus usually overestimated compared to its true, unknown value, which is termed the Rician bias in the following. Using the same remark as in the Gaussian case, that is, *E*(∑_*i*_ *w*_*i*_ *X*_*i*_^2^) = *μ* ^2^ + 2*σ* ^2^, it then seems natural to restore *x* as $\sqrt{\sum_{i}w_{i}x_{i}^{2}-2\sigma^{2}}$, the weights *w*_*i*_ summing to (1). The voxel value *x* can be restored as

(8) $$\text{NL}{\text{M}}_{R}\left(x\right)=\sqrt{\left(\sum_{x_{i}\in V}w_{i}x_{i}^{2}\right)-2\sigma^{2}},$$

where *σ* ^2^ is the noise variance. As noted by others in case if i.i.d random variables *X*_*i*_ and with *w*_*i*_ = 1/*n*, the term under the square root has a nonnull probability to be negative, which decreases when *n* is large. In such cases the restored value is set to zero. In practice, on real data, negative values are mainly found in the background of the images.

**Figure 2:**
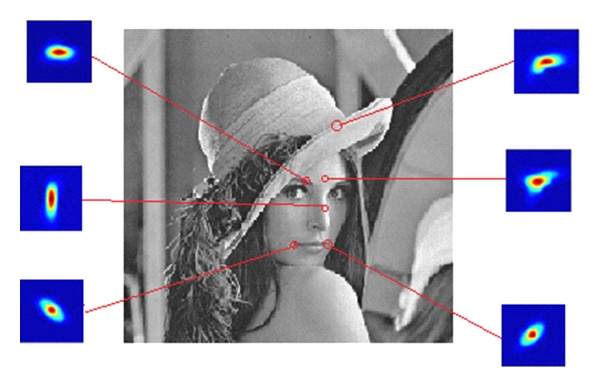
Steering kernels at different locations of the Lena image. The patch size is chosen to be 11 × 11.

 On the other hand, we should identify features that capture the underlying geometry of the image patches, without regard to the average intensity of the patches. For this, we make use of the data adaptive steering kernels developed by Takeda et al. [17]. In that work on Steering Kernel Regression (SKR), robust estimates of the local gradients are taken into account in analyzing the similarity between two pixels in a patch. The gradients are then used to describe the shape and size of the kernel. The steering kernel weight at the *j*th pixel in *i*th patch, which is a measure of similarity between the two pixels, is then given by

(9) $$w\left(i,j\right)=\frac{\sqrt{\det\left(C_{j}\right)}}{2\pi h^{2}}\exp\left\{-\frac{{\left(x_{i}-x_{j}\right)}^{T}C_{j}\left(x_{i}-x_{j}\right)}{2h^{2}}\right\},$$

where *h* is a global smoothing parameter also known as the bandwidth of the kernel. The matrix *C*_*j*_ denotes the gradient covariance formed from the estimated vertical and horizontal gradient of the *j*th pixel that lies in the *i*th patch. The 3 × 3 data-dependent steering matrix *C*_*j*_ can be defined as *C*_*j*_ = *h*(*H*_*i*_)^−1/2^, where *h* is a global smoothing parameter and *H*_*i*_ is a 3 × 3 covariance matrix based on the sample variations in a local neighborhood around sample *x*_*i*_. The weight *w*(*i*, *j*) is calculated for each location in the *i*th patch to form the weight matrix (or kernel). It is interesting to see that the weight matrix thus formed is indicative of the underlying image geometry. This fact is illustrated in Figure 2. Note that in each point of the weight matrix a different *C*_*j*_ is used to compute the weight, and hence, the kernels do not have simple elliptical contours.

 However, when dealing with nonstationary noise the use of a global noise variance across the image will lead to suboptimal results. To deal with this situation, local noise estimation should be introduced.

 Such estimation can be obtained by observing that the expectation of the squared Euclidean distance of two noisy patches as pointed out by Buades et al. is [12]

(10) $$\begin{array}{cc} d\left(N_{i},N_{j}\right) & =E{||u\left(N_{i}\right)-u\left(N_{j}\right)||}_{2}^{2}\\ & ={||u_{0}\left(N_{i}\right)-u_{0}\left(N_{j}\right)||}_{2}^{2}+2\sigma^{2}, \end{array}$$

where *u*_0_ is the noise-free image. Therefore, *d*(*N*_*i*_, *N*_*j*_) = 2*σ* ^2^ if *N*_*i*_ = *N*_*j*_. If we assume that each 2D patch in the volume has at least one patch equal to itself then the noise variance can be estimated as

(11) $$\sigma^{2}=\frac{\min\left(d\left(N_{i},N_{j}\right)\right)}{2}\;\forall j\neq i.$$

However, we found experimentally that this assumption is not normally met in real clinical conditions. In order to relax such an assumption we estimated the local variance as

(12) $$\sigma^{2}=\frac{\min\left(d\left(R_{i},R_{j}\right)\right)}{2}\;\;\forall j\neq i,\;R=u-\psi \left(u\right),$$

where the distance is calculated from a volume *R* computed as the subtraction of the original noisy volume *u* and the lowpass filtered volume *ψ*(*u*). We have found experimentally that the minimum distance in this case is approximately equal to *σ* ^2^ due to the removal of low-frequency information and the application of the minimum operator. 

 This Rician adapted filter removes bias intensity using the properties of the second-order moment of a Rice law. In fact, the second-order moment of a random variable *X* following a Rice distribution can be written as

(13) $$E\left(X^{2}\right)=\mu^{2}+2\sigma^{2}.$$

Consider a gray-scale image *y* = (*y*(*x*))_*x*∈*Ω*_ defined over a bounded domain *Ω* ⊂ *R* ^2^, and *y*(*x*) ∈ *R*_+_ is the noisy observed intensity at pixel *x* ∈ *Ω*. The weighting associated to the patch *P* is computed from the steering kernel:

(14) $$\begin{array}{cc} & W_{P}\left(i,j\right)\\ & \;=\frac{\sqrt{\det\left(C_{j}\right)}}{\sigma^{2}}\exp-\frac{1}{2}{\left(\frac{||y\left(x\right)-y\left(x_{i}\right)||}{\sigma^{2}}-\sqrt{2n-1}\right)}^{2}, \end{array}$$

where ||·|| denotes the Euclidean distance. *y*(*x*) : = (*y*(*x*_*k*_), *y*_*k*_ ∈ *B*(*x*)) ∈ *R* ^*n*^ is a vectorized image patch. *B*(*x*) is a $\sqrt{n}\times \sqrt{n}$ neighborhood centered at pixel *x*(*n* = 7). Δ(*x*) is a square neighborhood of *N* = |Δ(*x*)| pixels. *y*(*x*_*i*_) is a vectorized image patch such that *x*_*i*_ ∈ Δ(*x*). *σ* ^2^ is the noise variance assumed to be known or estimated. The final estimate is given by

(15) $$\text{IN}{\text{L}}_{\sigma ,n}y\left(x\right)=\frac{\sum_{P}W_{P}\left(i,j\right)y\left(x_{i}\right)}{\sum_{P}W_{P}\left(i,j\right)}.$$

The algorithm is divided in two identical separate steps, the image is scanned pixel per pixel. Let us denote by *P* the current reference patch which size is *n* × *n* (with *k* = 5) and *x*_*r*_ the current central pixel of *P*. The loop on the image is done on *x*_*r*_.

 This approach has two important benefits. On the one hand, it allows finding more similar patches with the same pattern but with different mean level compensating intensity inhomogeneities typically present on MRI data, and on the other hand, overestimation of the noise variance will be minimized in cases with unique patches in the search volume. Thus, the adaptive filter proposed will set the parameter *h* ^2^ equal to the minimum distance estimation as described in (11).

## 3. Experiments and Results

To evaluate and compare the proposed method with state-of-the-art methods, we did experiments on both synthetic and real medical images. To conduct the experiments on synthetic data, we use the standard MR images phantom of the brain obtained from the BrainWeb database [18]. The proposed algorithm was compared with the following recently proposed methods.

1. *NL-means: Nonlocal Means Image Denoising Method* [12]. The size of the patch and research window depend on the value of *σ*. The search window size used for experiments was 9 × 9 × 9, neighborhood size was 3 × 3 × 3, and value of the decay parameter *h* and *σ* were 0.4 *σ*, 20.
2. *NL-PCA: Nonlocal Principal Component Analysis Method* [19]. Local neighborhood size used for the experiments was 3 × 3 × 3. Other parameters are fixed to *α* = 2.1; *K* ^hard^ = *K* ^wien^ = 3; *n* ^hard^ = *n* ^wien^ = 15.
3. *DCT: Local Discrete Cosine Transform Method* [20]. The method decomposes the image into local patches, and denoises the patches with thresholding estimate in the DCT domain. The local patches of size used for the experiments was $\sqrt{N}=16\times 16$.
4. *Proposed Method*. The search window *n* for the experiments was 5 × 5 × 5.

For quantitative analysis of the denoising methods, we used the peak signal-to-noise ratio (PSNR), the structural similarity index matrix (SSIM).

**Figure 3:**
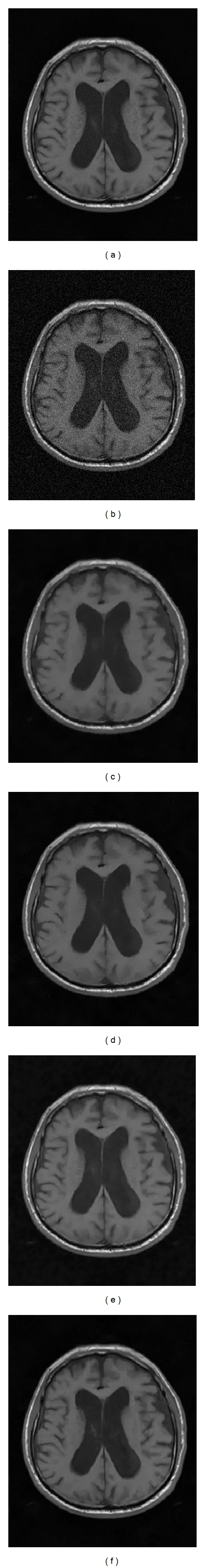
Denoising MRI with several methods: (a) original image; (b) original image corrupted by Rician noise of *σ* = 20; (c) denoised with NL-means method; (d) denoised with DCT method; (e) denoised with NL-PCA method; (f) denoised with proposed method.

**Figure 4:**
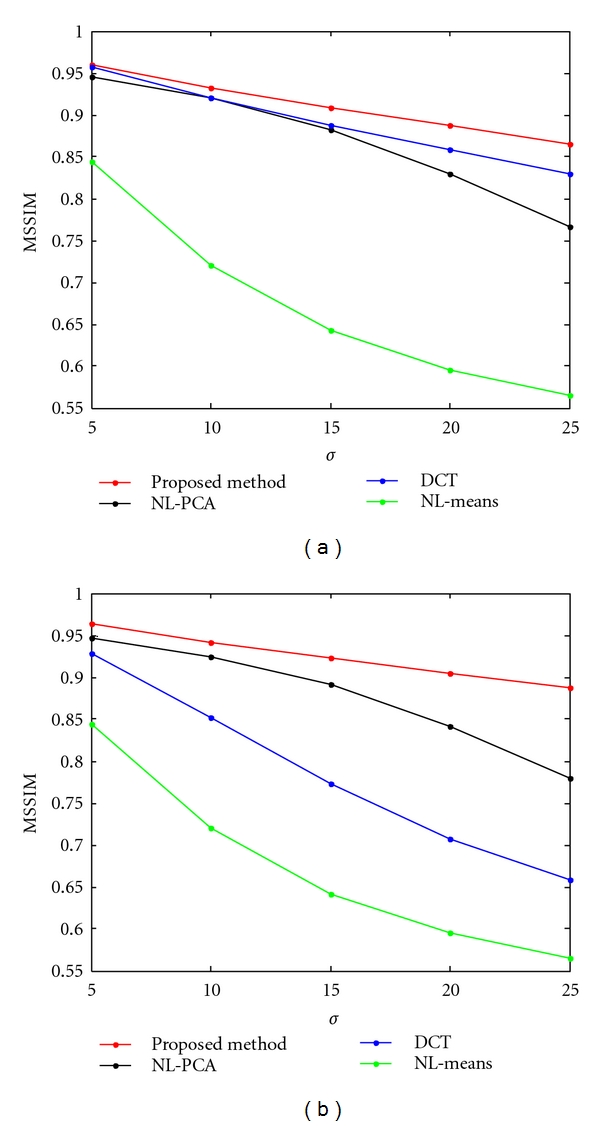
Comparative analysis of the proposed method with other methods based on PSNR, MSSIM for different values of the noise standard deviation.

Figure 3 displays the results of the image denoised with NL-means, NL-PCA, DCT, and proposed method. This experiment was conducted on the 2D slice of the synthetic images of the brain in the 3D environment after corrupting the image by uniform Rician noise with *σ* = 20. The proposed filter was executed using a neighborhood size for denoising as 13 × 13 × 13 and a neighborhood size for the local computation of range as 5 × 5 × 5. It can be observed from Figure 3 that the image denoised with the proposed method is closer to the original image than the images denoised with other approaches. The graph in Figure 4 shows the quantitative analysis of the proposed method with other recently proposed methods based on the similarity measures PSNR, MSSIM, respectively. This experiment was also conducted on the BrainWeb MR image with *σ* of the noise ranging from 10 to 30. All the methods with which the proposed method was compared are based on the Rician noise model. In the quantitative analysis, the background was excluded; that is, only the area of the image inside the skull was considered. It can be seen from the graph that the performance of the proposed method is best for each similarity measure.

**Figure 5:**
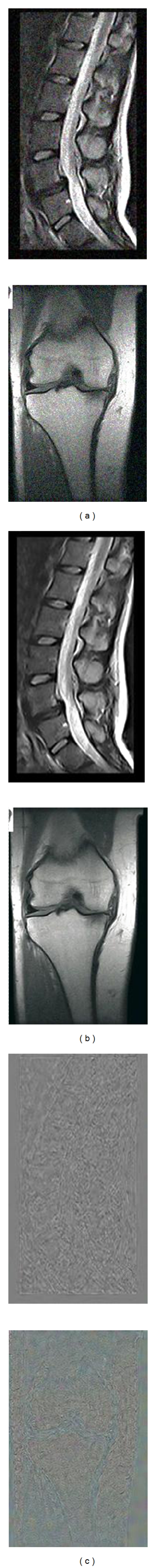
The denoising results obtained with the proposed filter. (a) The original noisy images (*σ* = 30); (b) the denoised images (PSNR is 38.9 and 37.7, resp.); (c) the differences of the images.

Figure 5 shows the extremely noisy data and we use the proposed method to remove the noise. The absolute value of the residuals of the filtering process clearly show the capabilities of the proposed approach on the extremely noisy data.

## 4. Conclusion

A new method to denoise the medical images by applying NL-means method is proposed in this paper. To demonstrate the efficiency of the proposed method, experiments were conducted on both simulated and real medical images. Comparative analysis with other recently proposed methods based on the similarity measures, PSNR, MSSIM, proves that the proposed method is superior to them in terms of image quality.
